# No Carbapenem Resistance in Pneumonia Caused by *Klebsiella* Species

**DOI:** 10.1097/MD.0000000000000527

**Published:** 2015-02-13

**Authors:** Josef Yayan, Beniam Ghebremedhin, Kurt Rasche

**Affiliations:** From the HELIOS Clinic Wuppertal, Witten/Herdecke University, Wuppertal, Germany.

## Abstract

*Klebsiella* species are a common cause of community- and nosocomial-acquired pneumonia. Antibiotic resistance to the class of carbapenem in patients with pneumonia caused by *Klebsiella* species is unusual. New studies report carbapenem resistance in patients with pneumonia caused by *Klebsiella* species.

This article examines, retrospectively, antibiotic resistance in patients with community- and nosocomial-acquired pneumonia caused by *Klebsiella* species.

The data of all patients with community- and nosocomial-acquired pneumonia caused by *Klebsiella* species were collected from the hospital charts at the HELIOS Clinic, Witten/Herdecke University, Wuppertal, Germany, within the study period 2004 to 2014. An antibiogram was created from all of the study patients with pneumonia caused by *Klebsiella* species. Sensitivity and resistance profiles were performed for the different antibiotics that have been consistently used in the treatment of patients with pneumonia caused by *Klebsiella* species. All demographic, clinical, and laboratory data of all of the patients with pneumonia caused by *Klebsiella* species were collected from the patients’ records.

During the study period of January 1, 2004, to August 12, 2014, 149 patients were identified with community- and nosocomial-acquired pneumonia affected by *Klebsiella* species. These patients had a mean age of 70.6 ± 13 (107 [71.8%, 95% CI 64.6%–79%] men and 42 [28.2%, 95% CI 21%–35.4%] women). In all of the patients with pneumonia caused by *Klebsiella* species, there was resistance to ampicillin (*P* < 0.0001). Many patients with pneumonia caused by *Klebsiella* species (75.3%) also showed resistance to piperacillin (*P* < 0.0001). However, no patients with pneumonia caused by *Klebsiella* species showed resistance to imipenem or meropenem (*P* < 0.0001).

Antibiotic resistance to the antibiotic class of carbapenem was not detected in patients with pneumonia caused by *Klebsiella* species.

## INTRODUCTION

*Klebsiella* is a human pathogen; it is a rod-shaped, gram-negative bacterium belonging to the family of Enterobacteriaceae. *Klebsiella* occurs everywhere in nature and in animals and humans alike. *Klebsiella* is mainly present in the intestinal flora, nose, and mouth in humans. *Klebsiella* can cause community- and hospital-acquired infections.^1^ There are different species of *Klebsiella: Klebsiella pneumoniae*, *Klebsiella oxytoca*, *Klebsiella granulomatis*, *Klebsiella terrigena*, and *Klebsiella planticola. K pneumoniae* and *K oxytoca* are most often detected in patients with pneumonia.^2^

The antibiotic penicillin was detected approximately 82 years ago. Since its discovery, penicillin has been increasingly used in the treatment of different kinds of infections. Gram-negative bacteria of the family of Enterobacteriaceae became resistant by the production of β-lactamases. The rapid increase of penicillinases in both gram-positive and gram-negative bacteria led to the development of cephalosporin β-lactam antibiotics.^3^ The group of carbapenem antibiotics were the only β-lactam agents effective against such broad-spectrum β-lactamase-producing bacterial strains. Whether the use of carbapenem was correct or unsuitable, this led to the development of carbapenem resistance in gram-negative bacteria.^4^ As a result of the increased use of carbapenem, 2 types of carbapenemase were soon detected: the metallo-β-lactamase and *K pneumoniae* carbapenemase. Meanwhile, there are 10 known variations of *K pneumoniae* carbapenemase, which differ from one another by 1 to 3 amino acid segments. The *K pneumoniae*–producing carbapenemase are not only multidrug resistant, they are also difficult to detect in a standard microbiology laboratory.^3^

Carbapenems are still an important class of antibiotics with broad-spectrum activity against many germs. Therefore, antibiotics of the carbapenem class are used as a reserve antibiotic as a last option for the treatment of many bacteria.^5^

Bacterial resistance to antibiotics can lead to life-threatening infections. Carbapenem-resistant *Klebsiella* species have been increasingly reported all over the world.^6,7^ The spread of carbapenem-resistant *Klebsiella* has led to an increase in the prevalence of *Klebsiella* species in the United States in the last 10 years.^8^ Carbapenem-resistant *Klebsiella* species were also reported in Europe, including Greece and Italy.^9^ Broad-spectrum antibiotics from the carbapenem group were spared from antibiotic resistance for a long time. The early and fast detection of patients with carbapenem-resistant *Klebsiella* species is necessary to avoid the spread of these highly resistant pathogens. The quick implementation of a strategy can help avert nosocomial outbreaks of *Klebsiella* species.^9^

In this study, an investigation was conducted to identify antibiotics that *Klebsiella* species were resistant in the last 10 years. Using the hospital database at the HELIOS Clinic, Witten/Herdecke University, Wuppertal, Germany, data were collected on all of the patients with *Klebsiella* species according to the International Classification of Diseases (ICD J15.0). The choice of the correct, effective antibiotic against *Klebsiella* species should shorten the duration of patients’ suffering and the length of their hospital stay, as well as reduce patient mortality.

## MATERIAL AND METHODS

### Patients

This quality-control observational study examined, retrospectively, the resistance to antibiotics in patients with diagnosed community- or nosocomial-acquired pneumonia triggered by *Klebsiella* species using data collected from hospital charts at the HELIOS Clinic, Witten/Herdecke University, Wuppertal, Germany, in the study period from January 1, 2004, to September 19, 2014. The study population with community- and nosocomial-acquired pneumonia initiated by *Klebsiella* species was mixed in terms of age. All patients over 18 years of age and who were detected to have community- or nosocomial-acquired pneumonia caused by *Klebsiella* species were included in the study. All of the patients with nosocomial-acquired pneumonia caused by *Klebsiella* species, but who were treated initially for other medical reasons in other departments, such as Internal Medicine and Surgery, were included in this study. Those with *Klebsiella* species that led to other infections, such as urinary infection, urosepsis, gastroenteritis, and meningitis, were excluded from this study. All of the patients examined at the Department of Neurology who had been suspected of having pneumonia caused by *Klebsiella* species were excluded from this study because of restricted access to their patient data.

### Definition of Pneumonia

Pneumonia is an acute inflammation of the lung, primarily affecting the alveoli, which is usually caused by infection from bacteria or viruses and less commonly other microorganisms.^10^ Typical clinical symptoms of pneumonia include cough, chest pain, fever, and difficulty breathing.^10^ The diagnosis of pneumonia is performed by x-ray examination and sputum culture.^10^

Community-acquired pneumonia caused by *Klebsiella* species is an acute infection of the lung parenchyma acquired from normal social contact in the community; this is in contrast to hospital-acquired pneumonia caused by *Klebsiella* species, which is acquired during hospitalization.^11^ The classification of pneumonia caused by *Klebsiella* species was made in each case, from 2004 to 2014, according to the latest edition of the ICD.^12^

### Tested Antibiotics

The sensitivity and resistance to the following antibiotics were tested against the *Klebsiella* species: ampicillin, piperacillin, ampicillin and sulbactam, piperacillin and tazobactam, cefuroxime, cefotaxime, cefepime, ceftazidime, imipenem, meropenem, ciprofloxacin, levofloxacin, co-trimoxazole, gentamicin, tobramycin, amikacin, tigecycline, tetracycline, and fosfomycin. The frequency of use of these antibiotics in clinical practice for the treatment of patients with pneumonia caused by *Klebsiella* species was recorded.

### Microbiology

The indication for the performance of microbiological examination was either routine or explicitly because of suspected respiratory infection. The secretion from the mouth-nasal cavity and trachea was obtained differently depending on the particular case; the commonly used methods applied were bronchoalveolar lavage, tracheal secretions, throat swabs, and sputum. The bronchoalveolar lavage was applied in the context of a bronchoscopy. The fiber-optic video bronchoscopies used were OLYMPUS type BF1T180 (Olympus Ltd, Hamburg, Germany) or high-resolution video bronchoscopy PENTAX type EPK-100p (Pentax Europe Ltd, Hamburg, Germany). In each case, about 20 mL of 0.9% saline solution were instilled under local anesthesia and aspirated through the fiber-optic bronchoscope again. The aspirate thus obtained was deposited in 3 different sterile, 40 mL specimen traps (Argyle Specimen Traps, Covidien Germany Ltd, Neustadt/Donau, Germany). Tracheal secretions were also collected by fiber-optic bronchoscopy through aspiration into sterile, 40 mL specimen traps (Argyle Specimen Traps, Covidien Germany Ltd, Neustadt/Donau, Germany). The throat swab was collected with a commercial cotton swab transport system (MEUS Srl, Piove di Sacco, Italy) by rotating the swab with slight pressure on the palatal arch of patients with suspected pneumonia. The recovery of sputum was performed by expectoration into a 30 mL sterile sputum collection tube (Salivette, SARSTEDT, Nümbrecht, Germany), which was then sent to the laboratory for analysis.

After the samples of sputum and tracheal and bronchial secretions were collected, the test samples were transported in suitable containers to the Institute of Microbiology and Virology. After propagation of the sputum in a sterile petri dish and testing against a dark background, a macroscopic evaluation was performed to categorize the samples as slimy, purulent, or bloody. Thereafter, a needle was used to separate the bronchial secretions and pus constituents of the saliva. Supportive shares of sputum and tracheal and bronchial secretions were used for microscopic examination. Microscopic examination was conducted after gram staining in 80- to 1000-fold magnification of at least 5 visual fields according to the criteria of Bartlett.^13^ More suspected diagnoses of the pathogen were expressed in the microscopic bacteriological examination than would be expected according to typical morphology and the microbiological infectiological quality standards. Determination was performed of the semiquantitative squamous epithelia, granulocytes, and microorganisms. After that, 3 solid culture media were applied for the cultivation of the most common aerobic, fast-growing microorganisms as a base culture. Columbia Agar with 5% sheep blood (Becton Dickinson, Heidelberg, Germany) was incubated at 37°C for 24 to 48 hours as a general culture medium for the growth and discovery of *Streptococcus pneumoniae*, *Streptococcus pyogenes*, *Staphylococcus aureus*, *Escherichia coli,* and *Shigella flexneri*. BBL CHROMagar Orientation medium (Becton Dickinson, Heidelberg, Germany) was used for the detection of Enterobacteriaceae. The tested Enterobacteriaceae were *Escherichia coli*, *Shigella*, *Klebsiella*, *Proteus mirabilis*, *Enterobacter* spp, *Citrobacter* spp, *Serratia marcescens*, *Salmonella*, and *Yersinia*. The medium BBL CDC Anaerobe 5% Sheep Blood Agar (Becton Dickinson, Heidelberg, Germany) was used for antimicrobial susceptibility testing for the general growth of anaerobes. The different *Klebsiella* species were identified using MALDI-TOF Biotyper mass spectrometry (Bruker Daltonik Ltd Life Sciences & Chemical Analysis, Bremen, Germany) and the National Reference Laboratory for Klebsiella Species, Department of Medical Microbiology, University of Kiel, Germany.^2^

BD Chocolate Agar (Becton Dickinson, Heidelberg, Germany) was used as a variant of blood agar for the isolation and cultivation of *Neisseria* and *Haemophilus* species, in which lysis of the erythrocytes was achieved through a brief heating of the agar at 80°C. The lysis caused hemin (factor X) and nicotinamide adenine dinucleotide (factor Y) to be released into the agar and subsequently metabolized by bacteria, resulting in the destruction of the hemolytics as well.

BD MacConkey Agar (Becton Dickinson, Heidelberg, Germany) was used as a selective medium for the detection of gram-negative bacteria.

BD Sabouraud Agar (Becton Dickinson, Heidelberg, Germany) was used for the cultivation and differentiation of fungi.

### Blood Cultures

Several blood cultures were employed to detect pathogens that propagate through the blood stream. First, skin was carefully disinfected with alcohol (72% ethanol and 10% propan-2-ol) by Bode Cutasept F (Bode Chemie Ltd, Hamburg, Germany). Then, with Braun Injekt single-use syringes (B. Braun Melsungen PLC, Melsungen, Germany), a minimum of 20 mL of blood was taken through venipuncture with a blood-collection needle (Safety-Multifly, SARSTEDT, Nümbrecht, Germany) and injected into 2 specific media—BACTEC Plus Aerobic/F and Plus Anaerobic/F medium (BD, Becton, Dickinson and Company, Heidelberg, Germany) and enriched soybean casein digest broth medium. After injecting the blood culture bottles with new needles, they were sent to the microbiology department where they were entered into a blood culture machine that incubated the specimens at body temperature. The blood culture instrument reported positive blood cultures with bacteria present; most cultures were monitored for 5 days, after which negative vials were removed.

No further investigation was carried out by polymerase chain reaction (PCR) in this study after the microbiological discovery of these germs. The application of PCR was used as a special requirement when no germ could be detected microbiologically in this study. When there was no microbiological evidence of bacteria, PCR was utilized in this study in cases of suspected β-lactamase-producing *K pneumoniae* after growth on the selective medium and a positive Hodge test. The target genes of carbapenemases belonged to the Ambler class A KPC, class B NDM-1, and class D OXA-48. According to genus and species, other genes of class B, such as IMP and VIM, were investigated. For the detection of β-lactamase-producing *K pneumoniae*, singleplex and multiplex PCRs were performed, which were mainly real-time PCRs.

### Laboratory

After the sample collection, the quantitative determination of C-reactive protein (CRP) in human serum and plasma (the normal value is less than 6 mg/L) was measured in lithium heparin SARSTEDT Monovette 4.7 mL (orange top) using a standard immunoturbidimetric assay on the COBAS 6000 INTEGRA system c 501 (Roche Diagnostics Ltd, Mannheim, Germany). The determination of the leukocyte count (normal range 4000–10,000/μL) in the blood was generally carried out as a routine part of blood counts after collection in EDTA Monovette 2.7 mL by flow cytometry using the Sysmex XE 2100 hematology analyzer (Sysmex Germany Ltd, Norderstedt, Germany).

### Comorbidities

The comorbidities were analyzed in patients with pneumonia caused by *Klebsiella* species. Comorbidity was considered the presence of 1 or more additional disorders existing simultaneously with the primary disease. The additional disorder may also be a behavioral or mental disorder.

Additionally, the length of the hospital stay was assessed in patients with pneumonia caused by *Klebsiella* species.

The number of deaths during hospitalization was determined in the study group. The survival analyses were completed using the Kaplan–Meier method; the number of days after discharge from the hospital that death occurred was calculated, and the total number of patients in the study group was considered.

### Ethics Statement

All of the patients’ data were anonymized prior to analysis. The Ethics Committee of the University of Witten-Herdecke in Germany approved this study. Due to the retrospective nature of the study protocol, the Ethics Committee of the University of Witten-Herdecke in Germany waived the need for written, informed consent.

### Statistical Analysis

The categorical data were expressed in proportion, while continuous data were expressed as a mean and standard deviation. The calculations were performed at 95% confidence intervals (CIs) for the sex difference of patients with pneumonia caused by *Klebsiella* species. A chi-square test for 2 independent standard normal variables of 3 probabilities was carried out for the detection of sensitivity and resistance to antibiotics used against *Klebsiella* species. A chi-square analysis was performed using the VassarStats Web site for statistical computation, created by Richard Lowry of Vassar College in Poughkeepsie, New York, USA.^14^ For the calculation of the *P* value using a 2 × 3 chi-square test, a contingency table was created containing up to 2 rows and 3 columns. The rows represented the amount of active substance of the antibiotics on antibiograms that was tested against *Klebsiella* species; the antibiotic substance ampicillin had the highest resistance profile when compared with the other antibiotic substances. The 3 columns were populated by numbers that categorized the *Klebsiella* species as sensitive, intermediary, or resistant to tested antibiotics, in order to calculate the results. Two-tailed tests were performed, and a *P* value of <0.05 was considered statistically significant.

## RESULTS

In the hospital database used in this study, 207 (2.9%, 95% CI 2.5%–3.3%) patients were found with pneumonia caused by *Klebsiella* species (ICD J15.0). This is compared to 6932 patients in all age groups with pneumonia caused by different types of germs, who had been treated at the HELIOS Clinic, Witten/Herdecke University, Wuppertal, Germany, during the study period of January 1, 2004, to August 12, 2014.

A total of 149 (2.1%, 95% CI 1.8%–2.4%) of 6932 patients with a mean age of 70.6 ± 13 years (107 [71.8%, 95% CI 64.6%–79%] men and 42 [28.2%, 95% CI 21%–35.4%] women) with pneumonia caused by *Klebsiella* species met the inclusion criteria for this trial. The male sex was more likely to suffer from pneumonia caused by *Klebsiella* species.

The patients were divided into categorical groups depending on the origin of their pneumonia caused by *Klebsiella*. These groups were community-acquired pneumonia, of which 73 patients belonged (49%, 95% CI 41%–57%); nosocomial-acquired pneumonia, of which 53 patients belonged (35.6%, 95% CI 27.9%–43.3%); and aspiration pneumonia, of which 23 patients belonged (15.4%, 95% CI 9.6%–21.2%).

Fifty-eight patients were excluded from this study. The reasons for the exclusion of these patients were that they had other infectious disease caused by *Klebsiella* species or that access to their patient data at the Department of Neurology was restricted. In addition, patients with pneumonia caused by *Klebsiella* species who were under the age of 18 and were treated at the Department of Pediatric and Adolescent Medicine were excluded.

The most-used antibiotics for the treatment of patients with pneumonia caused by *Klebsiella* species in this study were the piperacillin and tazobactam combination, the ampicillin and sulbactam combination, and imipenem (Table 1).

**TABLE 1:**
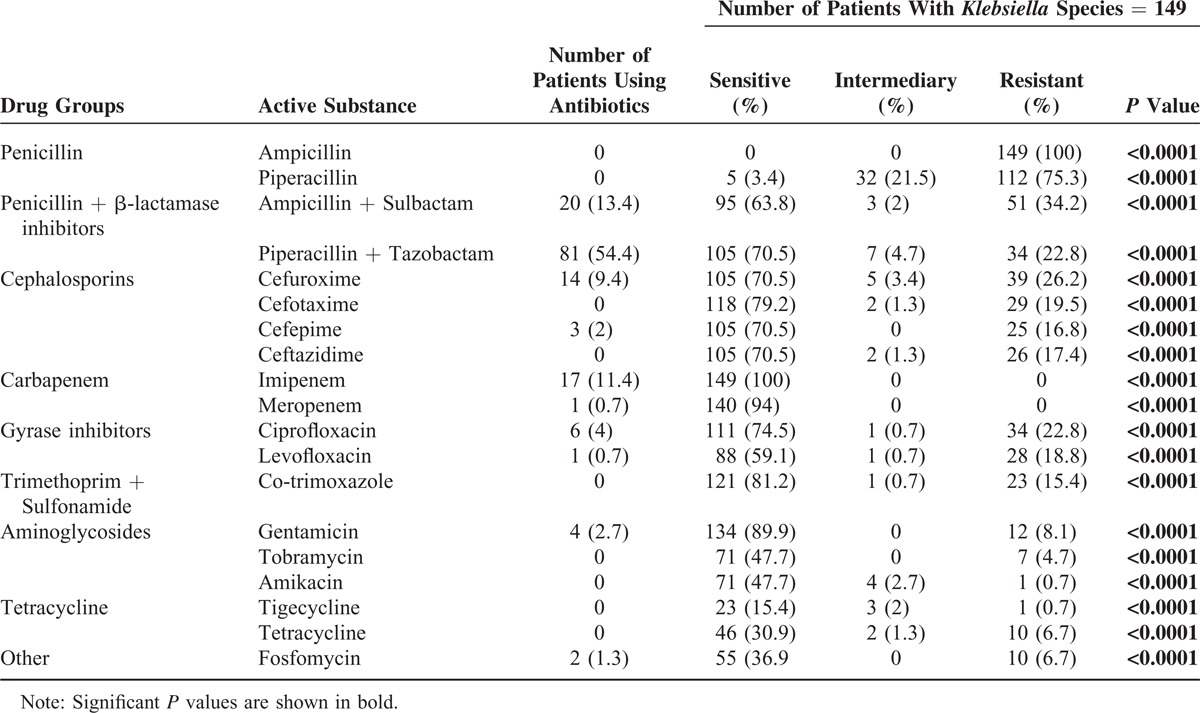
Drug Sensitivity and Drug Resistance in Different Drug Groups in Patients With Pneumonia Caused by *Klebsiella* Species

*Klebsiella* species had the highest resistance rate against the antibiotic group of penicillin in this study (Table 1). All of the patients with pneumonia caused by *Klebsiella* species showed resistance to ampicillin with statistical significance (*P* < 0.0001; Table 1). *Klebsiella* species also had a high resistance rate against piperacillin in this investigation (*P* < 0.0001; Table 1). *Klebsiella* species were less resistant against the groups of antibiotics including cephalosporins, gyrase inhibitors, the combination of trimethoprim and sulfonamide, and aminoglycosides (Table 1). However, *Klebsiella* species in patients with pneumonia were not resistant against the antibiotic group of carbapenem (*P* < 0.0001; Table 1). *Klebsiella* species in patients with pneumonia were all sensitive to imipenem and meropenem and had a relatively good sensitivity and resistance profile in the antibiograms against gentamicin (Table 1).

The tracheal secretions of patients with pneumonia caused by *Klebsiella* were sent to the Department of Microbiology at the HELIOS Clinic in Wuppertal, Germany, for further investigation into the bacteria present in the secretions (Table 2). Most of the discovered *Klebsiella* species were *K pneumoniae* and, less frequently, *K oxytoca* (Table 2).

**TABLE 2:**
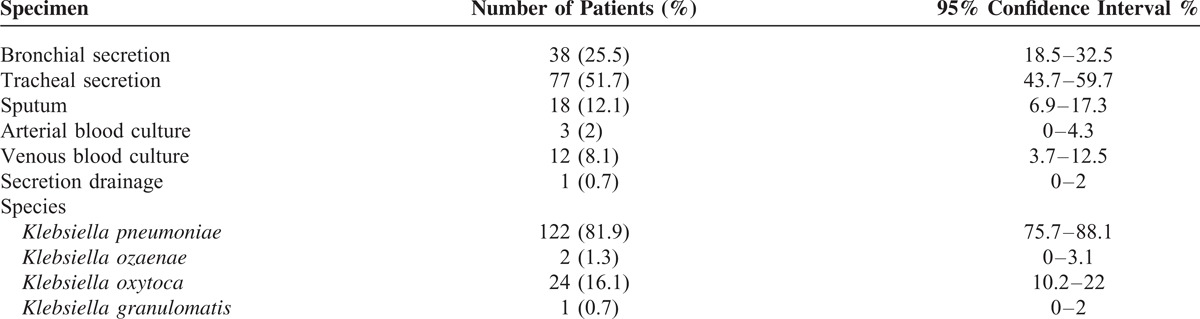
The Various Detection Methods and Species of *Klebsiella* in Patients With Community- and Hospital-Acquired Pneumonia

The amount of CRP in the serum and plasma of patients with pneumonia caused by *Klebsiella* species had a mean value of 112.9 mg/L ± 116.3 mg/L. The leukocyte count had a mean value of 13 338.2/μL ± 7240.1/μL in the blood of the patients with pneumonia caused by *Klebsiella* species.

Most discovered comorbidities were cardiac arrhythmias, sepsis, and acute renal failure in patients with pneumonia caused by *Klebsiella* species (Table 3 ).

**TABLE 3:**
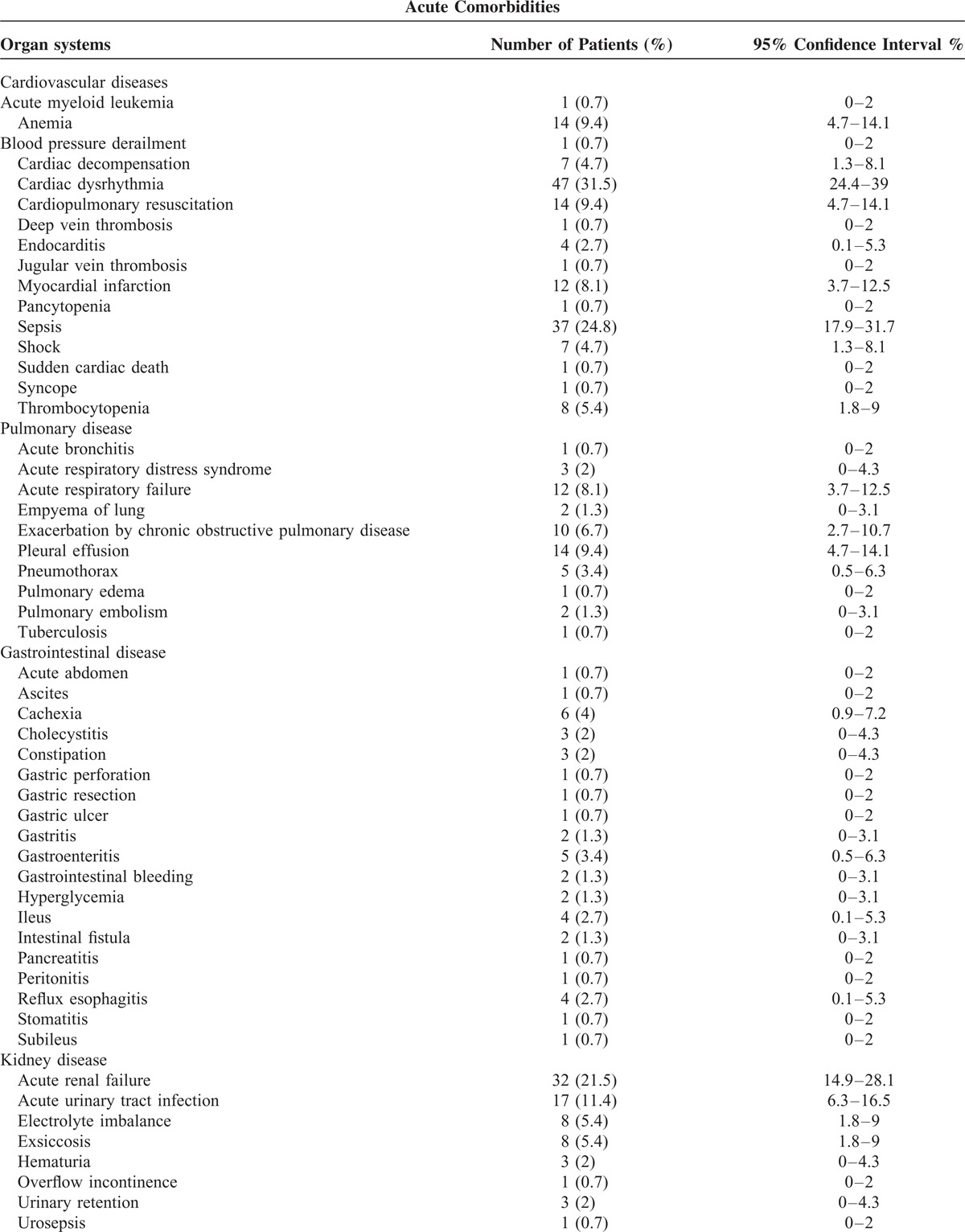
Acute Comorbidities in Patients With Pneumonia Caused by *Klebsiella* Species

**TABLE 3 (Continued):**
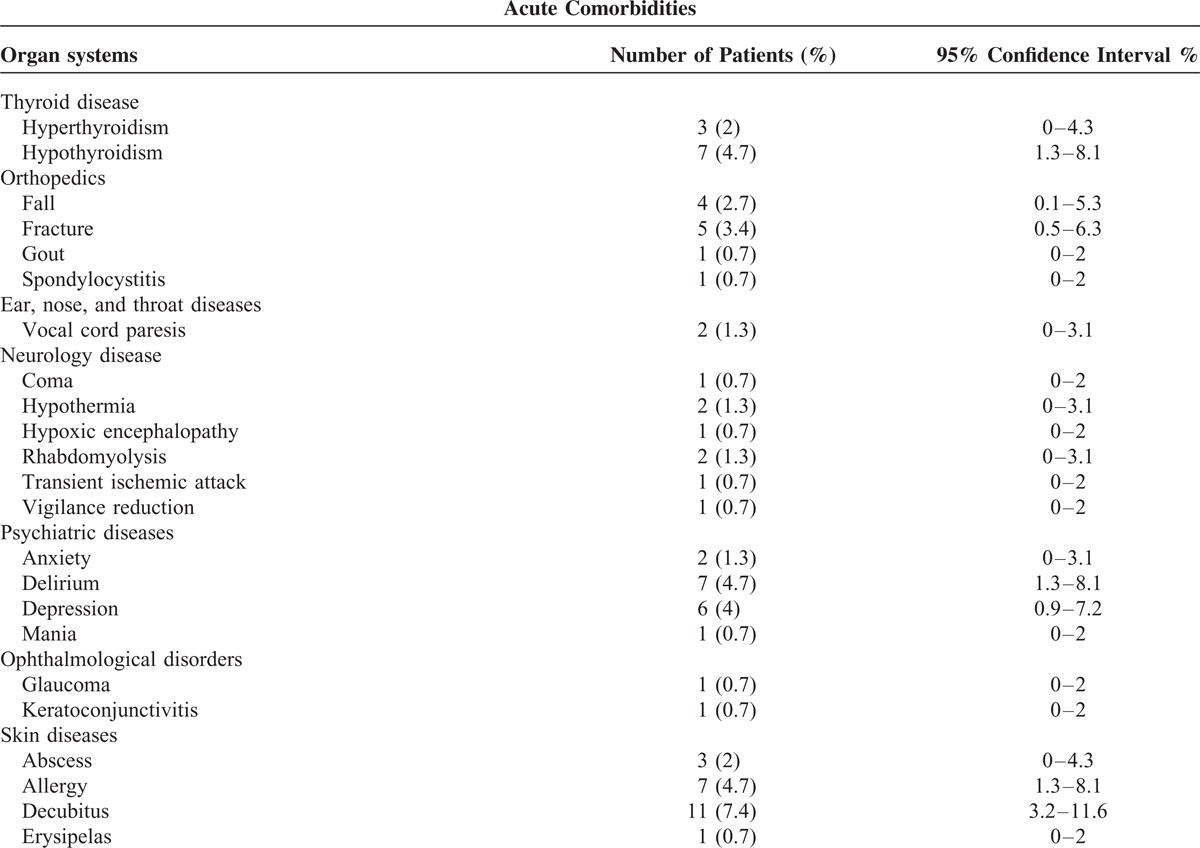
Acute Comorbidities in Patients With Pneumonia Caused by *Klebsiella* Species

The common chronic comorbidities were hypertension, coronary artery disease, and diabetes in patients with pneumonia caused by *Klebsiella* species (Table 4 ).

**TABLE 4:**
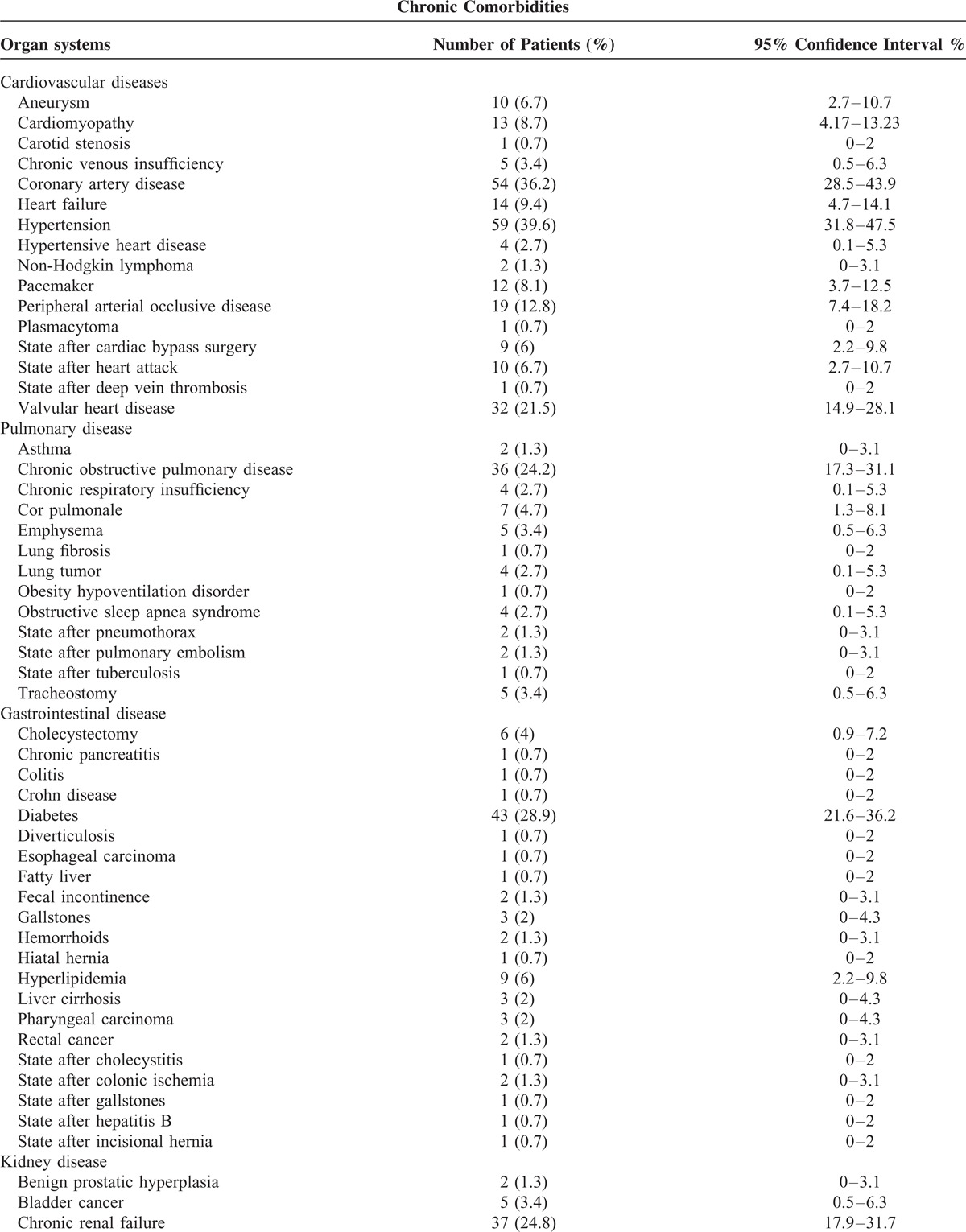
Chronic Comorbidities in Patients With Pneumonia Caused by *Klebsiella* Species

**TABLE 4 (Continued):**
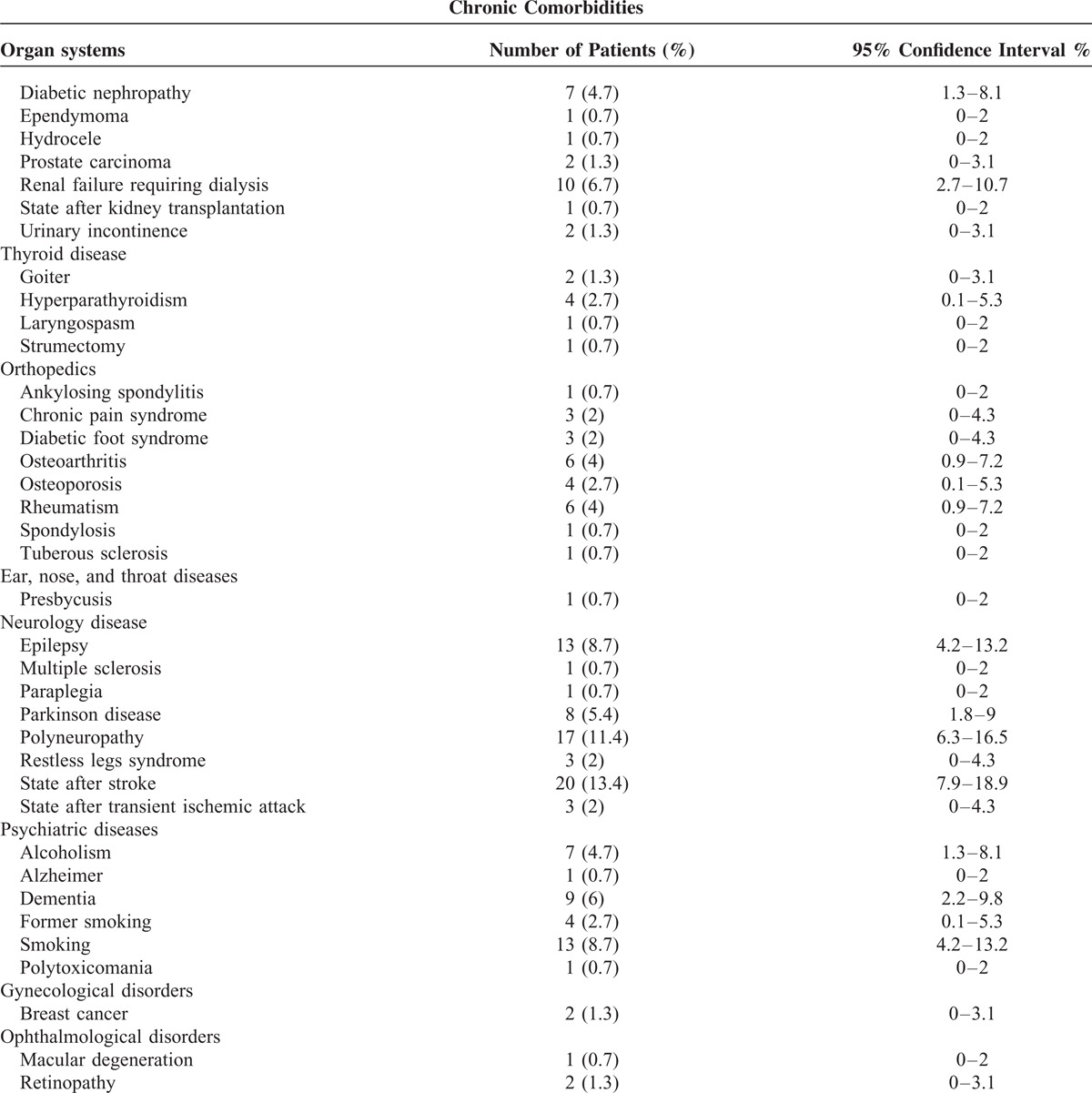
Chronic Comorbidities in Patients With Pneumonia Caused by *Klebsiella* Species

The length of the hospital stay of patients with pneumonia caused by *Klebsiella* had a mean of 22.9 ± 20 days.

There were 27 (18.1%, 95% CI 11.9%–24.3%) deaths associated with pneumonia caused by *Klebsiella.* Thus, the survival rate was 81.9% (95% CI 75%–88.7%) in patients with pneumonia caused by *Klebsiella* in this study.

## DISCUSSION

All of the patients with pneumonia caused by *Klebsiella* species showed resistance to ampicillin in this study. Similarly, an increased resistance profile was found for piperacillin in patients with pneumonia caused by *Klebsiella* in this study. Likewise, increased resistance was found for the combination of penicillin and β-lactamase inhibitors. From the results of this study, the conclusion was that *Klebsiella* species in patients with pneumonia had the highest resistance rate against the antibiotic group penicillin.

A worldwide study of several centers has previously shown an increased resistance to ampicillin and carbenicillin in patients with pneumonia caused by *Klebsiella*.^15^ Although carbenicillin was not tested in this study, resistance to other antibiotics was less frequent with isolates of *K pneumoniae* in the aforementioned global study.^15^

Another paper described ampicillin resistance in *K pneumoniae*.^16^ The study investigated the common resistance of pathogens isolated from clinical samples.^16^ The detection rate of *K pneumoniae* was high in that particular study.^16^

It is well known that the increased use of antibiotics leads to the development of resistance in bacteria to certain antibiotics. Due to this, a study assessed the influence of the augmented usage of piperacillin and tazobactam on antibiotic resistance.^17^ Despite the increased consumption of these antibiotics, the frequency of resistance of *K pneumoniae* to this antibiotic combination remained the same over the years.^17^

A high resistance to piperacillin and tazobactam was also reported in *K pneumoniae* in a study carried out much earlier.^18^ This study reports similar findings, with a high resistance to piperacillin and tazobactam also being observed in patients with pneumonia caused by *Klebsiella* species.

*K pneumoniae* can also cause aspiration pneumonia, but this type of pneumonia was not analyzed in this study. The effects of antibiotic therapy with ampicillin and sulbactam were compared with piperacillin and tazobactam in aspiration pneumonia in a previously published study.^19^ Better results were achieved with piperacillin and sulbactam than with ampicillin and sulbactam in the antibiotic therapy of patients with aspiration pneumonia in that study.^19^ No significant differences were found in the survival rate of patients with aspiration pneumonia between the 2 regimens of antibiotics.^19^ In the present study, a lower sensitivity and higher resistance were found in the antibiotic combination of ampicillin and sulbactam compared to the combination of piperacillin and tazobactam in the antibiogram of patients with pneumonia due to *Klebsiella* species.

The incidence of reduced sensitivity to cefuroxime was examined in clinical isolates of *K pneumoniae* from 2 different Danish regions.^20^ Eighty-three randomly selected clinical isolates of *K pneumoniae* with reduced susceptibility to cefuroxime were tested for cross-resistance to the production of β-lactamases in this Danish study. The frequency of resistance to cefuroxime and ciprofloxacin increased in 1990 from 5% to 15%. Two of the isolates of *K pneumoniae* were resistant to multiple drugs. Cross-resistance to ciprofloxacin showed, however, that other resistance mechanisms probably exist as penetration barriers in the Danish isolates of *K pneumoniae*. The sensitivity to ciprofloxacin decreased gradually with decreasing susceptibility to cefuroxime in patients with *K pneumoniae*.^20^ The same results were found with cefuroxime and ciprofloxacin in the present study. These 2 substances had low sensitivity in the microbiological testing of tracheal secretions from patients with pneumonia caused by *K pneumoniae*. The sensitivity to cefuroxime was more than two-thirds, and the resistance to cefuroxime was just over a quarter in this study. A similar result was obtained for ciprofloxacin in this study. It was noted that the resistance to ciprofloxacin in comparison to cefuroxime was slightly lower in this study.

One study examined the sensitivity of antimicrobial testing for antibiotics in patients with pneumonia due to *Klebsiella*.^21^ One of the tested antibiotics was cefotaxime. The result showed an increased resistance developed from 13.1% to 23.6% for cefotaxime in patients with pneumonia due to *Klebsiella*.^21^ The same result was obtained in the present study, with a similar rate of resistance of 19.5% to cefotaxime in patients with pneumonia caused by *Klebsiella* species.

The antibiotic cefepime belongs to the group of cephalosporins antibiotics. Cefepime is used in the empirical treatment of pneumonia in both gram-negative and gram-positive bacteria. One study examined the sensitivity of cefepime to microorganisms in an intensive care unit in 2 Iranian hospitals.^22^*Klebsiella* were commonly found among the Enterobacteriaceae in patients with nosocomial-acquired pneumonia.^22^ All isolated *Klebsiella* were completely resistant to cefepime in the Iranian study.^22^ The results of this study showed that cefepime was not the right choice for the empirical treatment of nosocomial-acquired pneumonia caused by *K pneumoniae*. A better sensitivity and resistance rate was found in the antibiogram for cefepime in the present study. In the present study two-thirds of the *Klebsiella pneumoniae* isolates were sensitive towards cefepime. The resistance to cefepime in this study was significantly lower at one-sixth of the patients with pneumonia caused by *Klebsiella* species compared to the previously mentioned study.

In another previously published study, a sensitivity of 14.4% for cefepime and 2.6% for ceftazidime was found in *K pneumoniae* carbapenemase producing Enterobacteriaceae.^23^ A significantly better sensitivity rate (70.5%) was found for these 2 antibiotics in this study.

All of the patients with pneumonia caused by *Klebsiella* species had a sensitive profile on their antibiogram in this study. A resistance to imipenem was not detected on the antibiogram of all of the patients with pneumonia caused by *Klebsiella* in this study. A similar result was found for the antibiotic meropenem of the same class of carbapenem. Almost all of the patients with pneumonia caused by *Klebsiella* were sensitive to meropenem, and none of the patients with pneumonia caused by *Klebsiella* had developed resistance to meropenem during the long observation period of 10 years in this study.

Bacteria-producing *K pneumoniae* carbapenemase have developed rapidly as a multidrug-resistant infection worldwide. These carbapenemase enzymes have been detected in *Klebsiella* species. These enzymes are capable of hydrolyzing a broad-spectrum of β-lactams, including penicillin, cephalosporins, carbapenem, and monobactam. The detection of these isolates with carbapenemase may be inconsistent; therefore, subsequent confirmatory tests are often necessary.^4^

Although sensitivity to imipenem and meropenem in *K pneumoniae* was found in the routine antibiotic sensitivity testing, these patients were positive in the further investigation of the PCR for carbapenemase-producing Enterobacteriaceae. When the patients with *K pneumoniae* carbapenemase were treated with imipenem or meropenem, clinical and microbiological failures were observed, as previous studies have reported.^24^ All of the *Klebsiella* species were isolated and identified using culture methods and MALDI-TOF or automated system Phoenix in this study, so the use of the PCR was not necessary.

In recent times, there are increased reports of carbapenemase-producing *K pneumoniae* in the medical literature. One study described the emergence and spread of carbapenemase-producing *K pneumoniae* in a Greek University Hospital. Using the PCR, isolates were studied coated with a carbapenem with minimum inhibitory concentration.^25^ An increased incidence of *K pneumoniae* carbapenemase with low incidence of β-lactamase-producing metallo-*Klebsiella* strains was isolated in the study that showed multidrug resistance.^25^ The most active substances were colistin, gentamicin, and fosfomycin on the isolates of carbapenemase-producing *K pneumoniae* in the study.^25^ The already high burden of antibiotic resistance created a major challenge for the Greek physicians in the treatment of the new carbapenemase-producing *K pneumoniae*.^25^

Studies have reported carbapenemase-producing *K pneumoniae* in other countries as well.^26^ It seems that carbapenemase-producing *K pneumoniae* spread over the world quickly. Reports from Norway and Sweden described the gram-negative infections for the first time in these countries. In these studies, the carbapenemase-producing *K pneumoniae* were also detected by PCR.^26^ With the increased detection of carbapenemase-producing *K pneumoniae* by PCR, the question was raised of whether or not to increase the routine use of PCR in the detection of these bacteria. Further studies are necessary to answer this question.

## STUDY LIMITATIONS

This study described the situation of *K pneumoniae* resistance in a single hospital, so the results cannot be generalized to other geographic locations. This study was unable to clarify whether or not the resistance rate of penicillin was due to the inappropriate use of these antibiotics in the treatment of patients over a long period. Also, a PCR was not always performed in all patients with *K pneumoniae.*

## CONCLUSIONS

All of the patients with pneumonia caused by *Klebsiella* species showed resistance to ampicillin. In addition, an increased resistance to piperacillin was detected in the study population with pneumonia caused by *Klebsiella* species. A decreased sensitivity and resistance was found for the antibiotic combinations of ampicillin with sulbactam and piperacillin with tazobactam. No patients with *K pneumoniae* showed resistance to the antibiotic class of carbapenem.
